# A Case Report of Acute Appendicitis and Intraperitoneal Abscess Causing Bowel Obstruction in the Setting of Ongoing Chemotherapy

**DOI:** 10.7759/cureus.103106

**Published:** 2026-02-06

**Authors:** Hannah Kruse, Abigayle Wyer, Jordan M Ross, Nathan Creel

**Affiliations:** 1 Surgery, Northeast Georgia Medical Center Gainesville, Gainesville, USA; 2 Research, Northeast Georgia Medical Center Gainesville, Gainesville, USA; 3 Trauma and Acute Care Surgery, Northeast Georgia Medical Center Gainesville, Gainesville, USA

**Keywords:** acute appendicitis, intraperitoneal abscess, mechanical bowel obstruction, small-bowel obstruction, taxane chemotherapy

## Abstract

Appendicitis and acute bowel obstruction represent two common complaints encountered by the acute care surgeon that can be overlooked in the setting of immunosuppressive chemotherapy, increasing the difficulty involved in effective treatment and minimizing mortality and morbidity. We report an instance of mechanical bowel obstruction secondary to acute appendicitis in a patient on chemotherapeutics for non-metastatic breast cancer. Early recognition, as well as an understanding of the possible impact of chemotherapeutic toxicity on the gastrointestinal tract, are imperative for morbidity reduction.

## Introduction

Bowel obstruction is a potentially life-threatening condition that requires prompt diagnosis and management. It accounts for approximately 15-20% of hospitalizations for acute abdomen, with 80% of cases attributed to postoperative adhesions [1,2]. Additional nonoperative etiologies include bowel herniation, malignancy, inflammatory bowel disease, motility disorders, and, rarely, volvulus [3,4]. The most common presentation of bowel obstruction is nausea, vomiting, abdominal pain, and distension, with loss of bowel function. Early diagnosis through CT scans is imperative for the reduction of morbidity and mortality secondary to electrolyte derangement from emesis and possible bowel perforation [5].

A less commonly observed cause of mechanical bowel obstruction is acute appendicitis. Presenting with a triad of right lower quadrant pain, fever, and anorexia, acute appendicitis accounts for approximately 300,000 yearly hospital visits in the United States [6]. Importantly, the presenting features are non-specific and may overlap with adverse responses to chemotherapeutic agents or manifestations of comorbid conditions, increasing the risk of diagnostic delay or inappropriate management of underlying appendicitis. Treatment of uncomplicated appendicitis most commonly consists of laparoscopic appendectomy, with limited morbidity and mortality in industrialized countries [7,8]. The relationship between acute appendicitis and bowel obstruction is traditionally limited to the formation of an adhesion, an Amyand hernia, or functional ileus [4], and mechanical obstruction is difficult to diagnose preoperatively [9].

This case study presents a complex clinical scenario of a patient undergoing chemotherapy who developed a mechanical bowel obstruction secondary to perforated appendicitis. Small bowel obstruction in the context of primary or metastatic cancers involving abdominal sites is relatively common; however, this case involves non-metastatic breast cancer. Taxane chemotherapies, including docetaxel, are commonly used for the treatment of both early-stage and metastatic breast cancer, but use is associated with varied toxicity that can impact patient health and quality of life [10]. The immunosuppressive effects of chemotherapy can mask classical signs and symptoms of intra-abdominal pathology, complicating both diagnosis and treatment, as well as increasing morbidity and mortality for the patient [11]. This report highlights the diagnostic challenges, ongoing management, and surgical considerations of acute bowel obstruction in the context of immunocompromised status due to ongoing cancer treatment. Importantly, surgeons should be aware that small bowel obstructions may occur in immunocompromised patients even in the absence of metastasis.

## Case presentation

The patient is a 74-year-old female with a past medical history significant for breast cancer without known metastasis. She presented to the emergency department with intractable nausea and vomiting. Her emesis started one week prior to her presentation and was described as dark/coffee ground in appearance by family members. Family reported that one week prior to the start of her emesis, she began a chemotherapeutic regimen involving cyclophosphamide and the taxane chemotherapeutic (TC) docetaxel. In addition to emesis, she also experienced symptoms of severe weakness, abdominal pain, and diarrhea. Her last episode of diarrhea was 12 hours prior to presentation, and she had not passed flatus since. On physical exam, her abdomen was distended but soft and focally tender in the lower quadrants and suprapubic region. The initial differential diagnosis included bowel obstruction, appendicitis, gastrointestinal (GI) tract, and gastroenteritis. Initial labs were significant for leukocytosis and acute kidney injury (Table 1).

**Table 1:** Lab values on presentation

Lab	Value	Reference Range
White Blood Cell	18.0 mg/dL	4.8-10.8 mg/dL
Blood Urea Nitrogen	44.0 mg/dL	5-23 mg/dL
Creatinine	5.07 mg/dL	0.6-1.0 mg/dL
Albumin	0.8 g/dL	3.4-5 g/dL
Lactic Acid	7.30 mmol/L	<2.00 mmol/L

**Figure 1:**
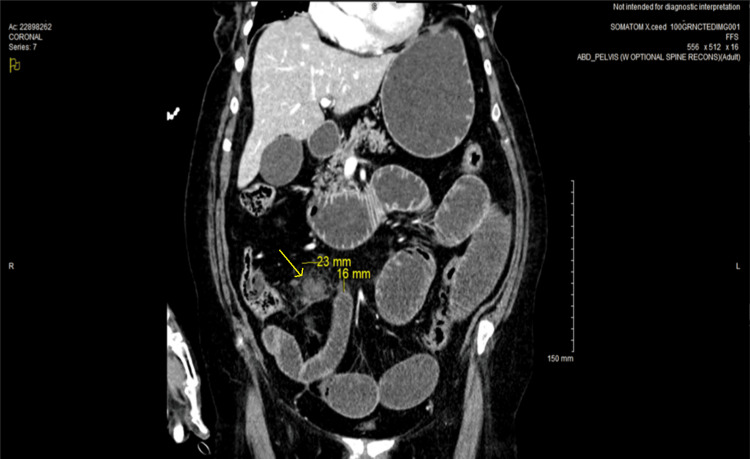
CT abdomen/pelvis with contrast on hospital day one CT imaging of the possible lead point and abscess in the ileocecal region (yellow arrow).

Initial CT imaging (Figure 1) demonstrated distended intestinal loops and a distended stomach, with a possible lead point in the ileocecal region contained within an area of local phlegmon. Based on these results, she was admitted to the surgical trauma ICU, and a nasogastric tube was placed to aid in decompression of the GI tract.

Clinical presentation declined significantly on hospital day one, and the patient experienced a significant worsening of kidney function and an increase in leukocytosis. The cause of the acute kidney injury was determined to be the small bowel obstruction, causing massive and intractable emesis, and a small bowel follow-through was initiated as an attempt at nonoperative management, given the patient's compromised wound healing status secondary to chemotherapy and lack of a clear transition point on CT imaging (Figure 1). Within two hours of initiating the small bowel follow-through, the procedure was aborted due to massive emesis of contrast, and the nasogastric tube was replaced. The decision was made to proceed to surgery, performing an exploratory laparotomy with washout.

Intraoperatively, the jejunum and proximal ileum were grossly dilated, with a decompressed distal ileum. There was no evidence of a clear transition point, stricture, or mass. The appendix was inflamed and gangrenous in parts with an adjacent abscess and reactive fluid drained intraoperatively with suction. Additionally, there was no evidence of metastatic lesions. An appendectomy with washout was performed, as well as the removal of the bowel from the ligament of Treitz to the rectum. The remaining bowel was determined to be viable and did not require resection. The removed gross specimen demonstrated appendiceal tissue with an area of possible perforation extending into yellow-tan soft tissue with yellow/green purulent material. Histopathology demonstrated acute appendicitis with periappendicitis, serositis, transmural abscess perforation, and perforation. The patient recovered successfully with an uneventful postoperative course and was discharged.

Six months into the postoperative period, the patient has not experienced immediate complications and has regained bowel function without additional need for surgery. At the time of this report, she and her family have elected to forgo any additional chemotherapeutic treatment.

## Discussion

Atypical presentations of common conditions in the setting of chemotherapeutics, as seen in this case, pose a diagnostic barrier to physicians, obscuring usual presentations. This is especially critical when timely diagnosis conveys a direct reduction of morbidity and mortality. In this patient, with a diminished physiologic reserve secondary to chemotherapy and a mixed clinical presentation, early identification was imperative to early action and subsequent recovery. The primary presentation of hematemesis, bilateral lower quadrant tenderness, diarrhea, and acute kidney injury in a non-peritonitic abdomen presents a broad range of diagnostic possibilities, which is only further obfuscated in the patient on chemotherapy.

Side effects of chemotherapy often include nausea, vomiting, and fever, overlapping with the presentation of both bowel obstructions and appendicitis. Previous reports identify metastatic cancer as a possible etiology of appendicitis and bowel obstruction [12-15], but uniquely, there was no evidence of metastasis in this patient.

The introduction of chemotherapeutic agents into the acute abdomen presents a diagnostic barrier. Certain chemotherapeutic agents, such as TCs, have been implicated in remodeling gastric mucosa and GI toxicity [10], which may have contributed to this patient's presentation. However, we caution that TC-induced gastric remodeling is a sparsely documented phenomenon, limited to animal studies, case reports, and a few meta-analyses, and further research would be required to formally implicate it in the pathogenesis of isolated appendiceal inflammation and subsequent perforation and abscess formation leading to small bowel obstruction in this patient [16-19]. Existing research demonstrates that TC use, independent of dose or toxicity level, may alter the GI tract mucosa mimicking high-grade dysplasia throughout the GI tract and may do so rapidly after TC administration [16]; however, this research does not delineate a timeline or if a toxic dose is required to incite these changes. Further research in this area is needed and could benefit the management of patients on chemotherapy.

Moreover, chemotherapeutic agents act as immunosuppressants in cancer patients, allowing for rapid disease progression and ultimately enabling atypical presentations of common diseases [11]. Rarely is acute appendicitis implicated in the development of a small bowel obstruction, though long-term sequelae may include adhesive disease and possible obstruction [4]. Even less frequently does acute appendicitis present with hematemesis and obstipation. Acute appendicitis and small bowel obstruction secondary to metastatic breast cancer is documented [12,14] though uncommon; however, this patient lacked metastatic disease that could have predisposed her to appendiceal perforation and obstruction. This case presents several practical clinical implications. The overlapping presenting symptoms between adverse chemotherapeutic response and intra-abdominal pathology, such as acute appendicitis and small bowel obstruction, highlight the need to maintain a broad differential diagnosis in immunocompromised patients. This may be especially relevant for oncological cases with no known metastasis. Immunosuppressants such as chemotherapeutics may exacerbate or obfuscate typical presentations, further necessitating broad differentials to reduce diagnostic delays and improve treatment and outcomes. It is important to tailor treatment strategies to the presenting individual, carefully weighing management against patient status.

## Conclusions

The true etiology of this patient's bowel obstruction is most likely a multifactorial immunocompromised state combined with the relative commonality of appendicitis. Currently, there is insufficient evidence to determine whether altered gastric mucosa secondary to TC could also be a factor, especially given the short exposure in this case. Additional investigation into the effects of TC on the GI tract are required to understand both the impact and timeline of downstream consequences. Such studies would serve to expedite the diagnostic process, improve treatment times, and reduce overall morbidity and mortality in a vulnerable population. Until then, this case highlights the imperative that any patient known to be receiving chemotherapy continue to be approached with a broad differential to reduce the time to necessary surgery and, subsequently, morbidity and mortality in a vulnerable population.
